# Role of Training Paramedical Staff for Cervical Cancer Screening via Human Papillomavirus Self-Sampling in Remote Mountainous Regions: A Pilot Feasibility Study

**DOI:** 10.7759/cureus.102046

**Published:** 2026-01-22

**Authors:** Nilanchali Singh, Vikas Raj Jalagam, Sushmitha Somagattu, Sadaf Kunwar, Pranay Tanwar

**Affiliations:** 1 Obstetrics and Gynaecology, All India Institute of Medical Sciences, New Delhi, New Delhi, IND; 2 Laboratory Oncology, All India Institute of Medical Sciences, New Delhi, New Delhi, IND

**Keywords:** cervical cancer, difficult terrains, hpv, paramedical staff, screening

## Abstract

Background

Cervical cancer is the second most common cancer among women in India and a leading cause of cancer-related deaths in low- and middle-income countries. Despite the availability of effective screening methods, coverage remains low in India due to limited awareness, logistical barriers, and poor access, especially in remote regions. The World Health Organization recommends human papillomavirus (HPV) DNA testing as the preferred screening method, and self-sampling has emerged as a promising strategy to increase participation. This study aimed to evaluate the feasibility and acceptability of HPV self-sampling for cervical cancer screening through trained paramedical staff in a community-based pilot study conducted in a remote mountainous region of Ladakh, India.

Methodology

This was a pilot, observational feasibility study conducted during a community-based health camp in Kargil, Ladakh. Paramedical staff (auxiliary nurse midwives) were trained through demonstrations and videos to counsel women on HPV self-sampling. Women were educated and offered self-sampling kits (Digene HC2 High-Risk HPV DNA, Qiagen). Samples were collected on-site and transported to New Delhi for testing. Reports were shared via mobile phone and followed up by local gynecologists.

Results

Of the 50 women counseled, 47 (94%) consented to HPV self-sampling. One tested positive (2.1%) for high-risk HPV and was referred for further evaluation. The high acceptability indicates the feasibility of this approach in remote areas.

Conclusions

This pilot study demonstrates that training paramedical staff for HPV self-sampling facilitated by trained paramedical staff appears to be a feasible and acceptable approach for cervical cancer screening in remote settings. This approach can bridge screening gaps and support national efforts toward cervical cancer elimination.

## Introduction

Cervical cancer ranks as the fourth most common cancer among women globally and the second most common in India. It is a significant contributor to cancer-related deaths in low- and middle-income countries (LMICs) [1]. According to Globocan 2020, there were 604,100 new instances of cervical cancer worldwide in 2020, and the disease was responsible for 341,831 fatalities. In 2020, cervical cancer accounted for 18.3% (123,907) of new cases and 9.4% of all cancer cases in India. It continues to be one of the most common malignancies in India and a major contributor to cancer-related mortality among women in LMICs [2], accounting for one-fourth of all cervical cancer mortalities worldwide [3]. Cervical cancer screening is a crucial secondary prevention measure due to the substantial disease burden, particularly in LMICs like India, where it remains a leading cause of cancer-related deaths among women. Population-based screening is an important secondary preventive measure for cervical cancer [1]. While around 68-84% of women are screened in developed countries, the rate is only 2.5-5% in India [4]. Despite available screening methods, their effectiveness in India is limited by negative attitudes, poor practices, and a lack of awareness regarding preventive measures.

According to the World Health Organization (WHO) program for cervical cancer elimination, new guidelines recommend human papillomavirus (HPV) DNA testing as the preferred screening method over visual inspection with acetic acid (VIA) and cytology [5]. HPV DNA testing is simpler and more cost-effective compared to VIA and cytology, making it the most commonly used screening method globally.

Self-sampling has emerged as a preferred option among participants in screening programs [6]. Giving cervical cancer screening to trained community or paramedical professionals greatly increases uptake and feasibility, according to recent studies from India and other LMICs. Even in settings with limited resources, telecounseling and structured training for frontline health workers, such as ASHA employees and auxiliary nurse midwives, have demonstrated high acceptability and confidence in counseling women for HPV self-sampling. These strategies assist in removing obstacles pertaining to literacy, cultural stigma, and lack of procedural confidence, especially in marginalized communities [7].

This can increase the number of women being tested and support the goal of screening 70% of eligible women by 2030. Barriers to self-sampling include illiteracy, lack of confidence in performing the procedure correctly, and concerns about pain and anxiety [8]. Community health workers and paramedical staff can bridge this gap by counseling women in the community about the technique and addressing potential barriers. This pilot study analyzes a cervical screening strategy involving training paramedical staff in a mountainous region in Ladakh, India, with challenging topography, to counsel women in communities about HPV self-sampling.

## Materials and methods

This was a pilot, observational feasibility study conducted during a community-based health camp in Kargil, Ladakh. The health camp was organized by a non-profit organization in a remote mountainous region. The study evaluated cervical cancer screening using HPV self-sampling facilitated by trained paramedical staff.

Training the staff

The paramedical staff (auxiliary nurse midwives) were trained by the physician before starting the camp. Training was done by demonstration and video demonstration, followed by a question-and-answer session. Thereafter, women attending the camp were counseled about HPV self-sampling by trained paramedical staff. The women were educated about the procedure by showing videos and answering their queries. They were provided with a secluded, safe, and clean space for self-sampling.

Self-sampling method

The self-sampling technique involves obtaining one’s own vaginal sample using a kit (Digene HC2 High-Risk HPV DNA, Qiagen, Hilden, Germany) provided at the camp. The kit included a cervical brush and a vial containing transport medium. Ideally, sampling can be done at any private location in the comfort of one’s home. In this study, self-sampling was conducted on-site at the camp.

Sample collection and transportation

**Figure 1:**
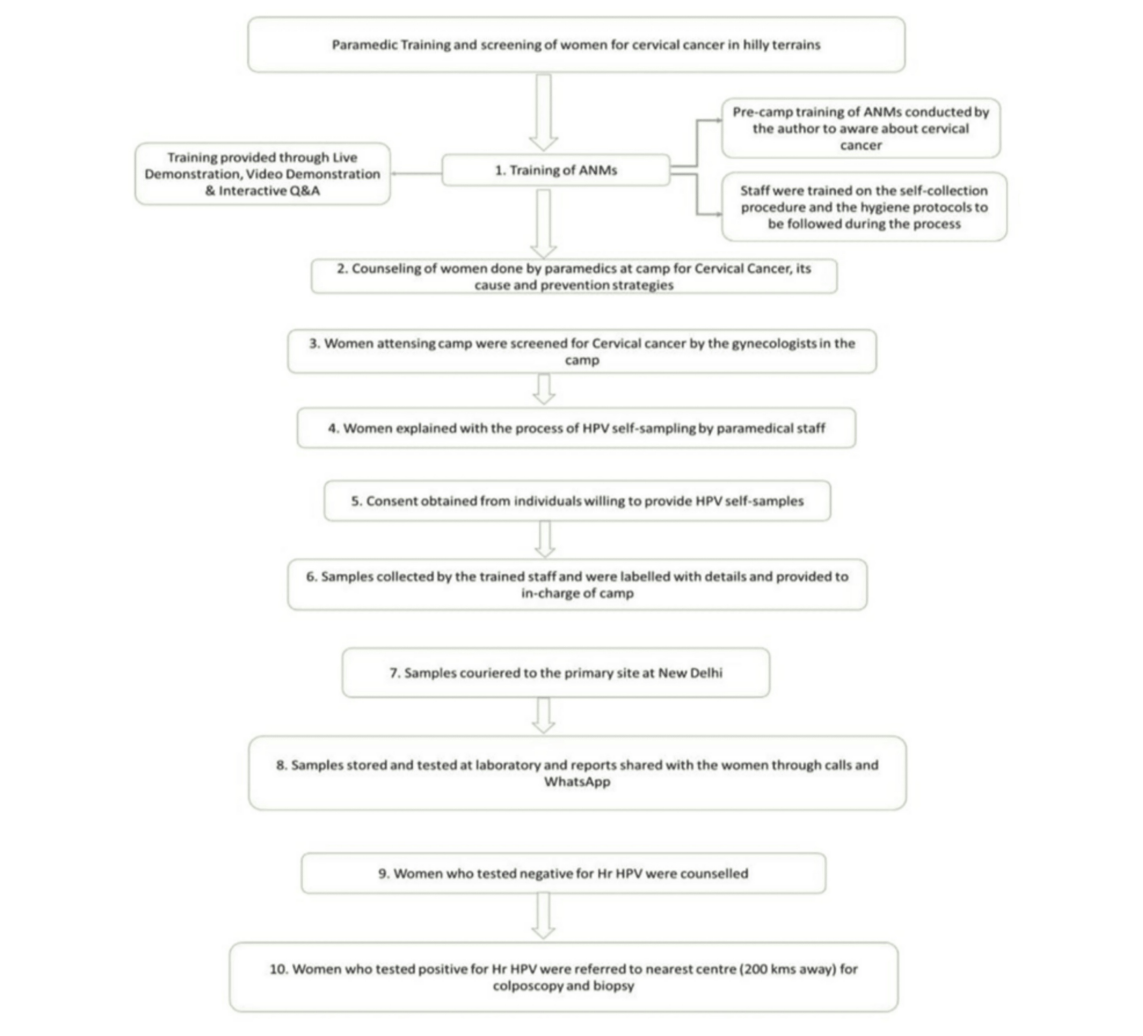
Flowchart illustrating the training of auxiliary nurse midwives (ANMs) and cervical cancer screening via human papillomavirus (HPV) self-sampling.

Collected samples were handed over to paramedical staff by the patients, and the samples were properly labeled with patient details and phone numbers. Collected samples were stored and transported at room temperature. Thereafter, the samples were transported to the primary site at New Delhi for testing by courier service (Figure 1).

Data collection

Demographic details such as age, address, and contact information were recorded. Their experiences and reasons for refusal, if any, were recorded. The sample collection was done over a period of three days.

Sample testing

All collected samples were received via courier at the laboratory facility at the primary study site in New Delhi. Upon receipt, they were systematically documented and stored until further processing. The samples were then tested for high-risk HPV using the Hybrid Capture II method.

Report sharing and post-test counseling

The gynecologists posted in the District Hospital, Kargil, were also trained to follow up patients and conduct post-test counseling. After testing, the reports were shared over mobile phone and over WhatsApp. The reports were also shared with the gynecologist posted at the center. Post-test counseling was done by the gynecologist posted in the District Hospital, Kargil. Those who tested negative were counseled. Those who tested positive were referred to the nearest center, around 200 km from the testing site, for colposcopy and biopsy.

## Results

As part of the cervical cancer screening initiative, approximately 50 women were approached and educated about self-sampling for HPV testing at the camp. Of these, 47 women consented to self-sampling and successfully provided self-collected samples at the camp, resulting in an acceptance rate of 94%, demonstrating good acceptability of the method. Table 1 presents an overview of pre-camp training of auxiliary nurse midwives for cervical cancer and HPV self-sampling. The demographic characteristics and participation outcomes of the screened women are summarized in Table 2. The median age of the participants was 35.5 years, with the majority being premenopausal. The samples were tested for high-risk human papillomavirus (hrHPV), the primary causative agent of cervical cancer. Among the 47 samples, one patient tested positive for hrHPV (2.1%), while the remaining 46 (97.9%) tested negative (Table 3). The reports were provided to the patients telephonically and by WhatsApp. The hrHPV-positive patient was promptly informed, counseled, and scheduled for further evaluation and appropriate clinical management, including colposcopy with/without biopsy, ensuring timely follow-up. Women testing negative for high-risk HPV were counseled and advised to repeat the test after five years.

**Table 1:** Overview of pre-camp training of auxiliary nurse midwives for cervical cancer and human papillomavirus self-sampling.

Characteristic	Details
Number of staff trained	5
Training conducted by	Author
Mode of training	Live demonstration, video demonstration, interactive Q&A
Training duration	2 hours
Training content	Cervical cancer and its causes (human papillomavirus), risk factors, symptoms, and prevention strategies such as vaccination, self-sampling procedure, and patient follow-up
Educational tools used	Educational video, sampling kits, flowcharts, and posters
Assessment method	Informal Q&A, observation through mock sessions
Confidence level post-training	The majority rated as “Confident” or “Very Confident”

**Table 2:** Demographic characteristics and participation results (N = 47).

Variable	Value/Frequency (n = 47), n (%)
Education	
No formal schooling	27.7%
Primary	38.3%
Secondary	17.0%
Graduate	8.5%
Postgraduate	8.5%
Occupation	
Employed	12.8%
Unemployed	87.2%
Parity	
Nulliparous	10.6%
Para 1	80.9%
Para 2	6.4%
Para 3	2.1%
Socioeconomic status	
Upper class	4.3%
Middle class	89.4%
Lower class	6.4%
Menopausal status	
Pre-menopausal	80.9%
Post-menopausal	19.1%
Smoking status	
Smoker	10.6%
Non-smoker	89.4%

**Table 3:** Results of cervical cancer screening by human papillomavirus (HPV) self-sampling in women.

Characteristic	Value
Age in years, median	35.5
Number of women counseled	50
Prior awareness of cervical cancer and HPV	12 (24%)
Number of women giving consent for self-sampling	47 (94%)
Reason for refusal: will get tested on a later date	1 (2%)
Don’t think the test is important	1 (2%)
Cultural issues	1 (2%)
Number of women satisfied with the self-sampling process	47 (94%)
Women testing positive for high-risk HPV	1/47 (2.1%)
Women testing negative for high-risk HPV	46/47 (97.9%)
Number of women advised for follow-up	1/47 (2.1%)

## Discussion

This pilot study demonstrates a unique approach to cervical cancer screening in a remote, hilly terrain area in LMICs, where training paramedical staff in cervical cancer awareness, screening, and self-sampling methods can be an effective strategy. This camp-based approach evaluated HPV DNA testing via self-sampling. Current WHO guidelines recommend HPV DNA testing as a preferred method for cervical cancer screening. Therefore, the strategy used in this camp is feasible and acceptable. Supporting evidence indicates that HPV self-sampling has good acceptability among Indian women and shows good agreement with samples collected by health personnel [9]. The acceptance rate of 94% observed in this study is comparable to, and in some cases higher than, rates reported in community-based self-sampling studies conducted in urban slums and rural plains of India, where acceptance has ranged between 58% and 98% [9]. This finding suggests that geographical remoteness alone does not negatively influence acceptability when appropriate counseling, privacy, and support are provided through trained healthcare personnel [8]. A community-based interventional study by Mishra et al. found acceptance rates of self-sampling to be 97% in urban slum areas and 98.8% in rural areas [9]. Convenience, privacy, cost-effectiveness, and ease of use are key factors driving the widespread adoption of self-sampling in communities.

This study was conducted in Leh Ladakh, a hilly terrain region in northern India. Topography significantly impacts access to healthcare facilities [10]. Despite growing evidence supporting HPV self-sampling, data from remote mountainous regions such as Ladakh are extremely limited. Harsh climatic conditions, sparse population distribution, long travel distances, and limited specialist availability pose unique challenges to organized cervical cancer screening in these regions. Evaluating innovative, camp-based, paramedical staff-led self-sampling models in such settings is therefore crucial to inform scalable national strategies [8]. People living in hilly terrains like Ladakh face challenges such as limited transportation and very low temperatures. Consequently, the population in these areas is often underscreened compared to other regions. Health camps offering specialist services are a cost-effective way to bridge the gap in healthcare coverage in these hard-to-reach hilly areas [11]. Hence, this camp-based study was conducted to screen women in Ladakh who are difficult to reach. Furthermore, samples collected from the women can be easily transported to testing facilities, and follow-up care for Hr-HPV positive women can be easily provided at care centers. This approach is cost-effective and time-saving, as the rough terrain makes it difficult for locals to visit tertiary care centers in other states. Therefore, self-sampling can enhance screening efforts and boost awareness of cervical cancer.

Strong evidence supports the usefulness of self-sampling in increasing participation among hard-to-reach women in screening programs [12]. However, challenges such as a lack of proper knowledge and technique and fear and anxiety about the procedure persist. Key barriers include a lack of confidence and cultural implications related to sexual behavior. These barriers can be overcome through the involvement of physicians and other members of the healthcare community in counseling and educating individuals about these issues [13]. Accordingly, paramedical staff received training in the self-sampling procedure and subsequently provided counseling to women attending the camp.

Strengths and limitations

The strength of this study lies in its innovative approach, where paramedical staff were actively involved in cervical cancer screening. If this camp-based screening strategy proves effective, it can potentially bridge the screening coverage gap in difficult-to-reach hilly areas due to its cost-effectiveness. This strategy is particularly beneficial in challenging terrains.

The limitation of this study is the small sample size, as fewer women were screened in the camp due to its pilot design. Additionally, it is a single-center study conducted in Ladakh. Cultural values, community norms, acceptability, and practicality may vary across the nation, necessitating evaluation in other centers.

## Conclusions

This study assesses the feasibility and effectiveness of self-sampling for cervical cancer screening in a remote, hard-to-reach hilly region. Physicians trained paramedical staff on the self-sampling procedure, who, in turn, counseled women attending the camp regarding the technique and addressed potential minor concerns. It is important to note that this study was designed to explore feasibility and acceptability rather than to measure clinical effectiveness or long-term outcomes. As a small, single-center pilot study, the findings should be interpreted with caution. However, the results provide useful early evidence that such a model can work in challenging settings with limited resources. The findings highlight a pragmatic and scalable screening strategy that leverages paramedical personnel to reach underserved populations in geographically challenging areas, hence bridging the gap.
